# An Eye on the First Surgical Side: Appreciating the Potential Impacts of a Second DBS Lead on Ipsilateral Symptoms

**DOI:** 10.5334/tohm.918

**Published:** 2024-07-04

**Authors:** Ariane Veilleux Carpentier, Jason L. Chan, Joshua K. Wong, Michael S. Okun

**Affiliations:** 1 Norman Fixel Institute for Neurological Diseases, University of Florida, Gainesville, Florida, USA; 2 Department of Neurology, University of Florida, Gainesville, Florida, USA

**Keywords:** deep brain stimulation, staged surgeries, microlesion effect, essential tremor, patient selection, ipsilateral

## Abstract

**Clinical Vignette:**

A 63-year-old man with severe essential tremor underwent staged bilateral ventralis intermedius (Vim) deep brain stimulation (DBS). Left Vim DBS resulted in improved right upper extremity tremor control. Months later, the addition of right Vim DBS to the other brain hemisphere was associated with acute worsening of the right upper extremity tremor.

**Clinical Dilemma:**

In staged bilateral Vim DBS, second lead implantation may possibly alter ipsilateral tremor control. While ipsilateral improvement is common, rarely, it can disrupt previously achieved benefit.

**Clinical Solution:**

DBS programming, including an increase in left Vim DBS amplitude, re-established and enhanced bilateral tremor control.

**Gap in Knowledge:**

The mechanisms underlying changes in ipsilateral tremor control following a second lead implantation are unknown. In this case, worsening and subsequent improvement after optimization highlight the potential impact of DBS implantation on the ipsilateral side.

**Expert Commentary:**

After staged bilateral Vim DBS, clinicians should keep an eye on the first or original DBS side and carefully monitor for emergent side effects or worsening in tremor. Ipsilateral effects resulting from DBS implantation present a reprogramming opportunity with a potential to further optimize clinical outcomes.

**Highlights:**

This case report highlights the potential for ipsilateral tremor worsening following staged bilateral DBS and provides valuable insights into troubleshooting and reprogramming strategies. The report emphasizes the importance of vigilant monitoring and individualized management in optimizing clinical outcomes for patients undergoing staged bilateral DBS for essential tremor.

## Clinical Vignette

A 63-year-old right-hand-dominant man presented for deep brain stimulation (DBS) programming of his second contralateral lead following staged bilateral thalamic DBS, which was implanted to address severe, medication-refractory essential tremor (ET). The patient had been experiencing action tremor in both upper extremities for approximately 40 years prior to presentation. As the tremor progressed, it began to involve his head and voice, and significantly impacted his quality of life. He was unable to perform manual tasks, leading him to stop working and ultimately decide to move into an assisted living facility.

**Figure 1:**
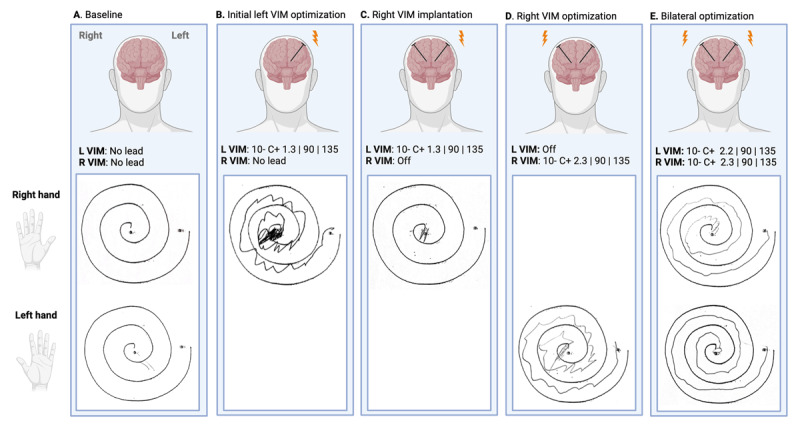
**Archimedes spirals evolution. A:** Prior to staged bilateral Vim DBS, the patient was unable to draw Archimedes spirals with either hand due to severe tremor. **B:** After initial optimization of the left Vim DBS, his right-hand tremor improved. **C:** After right Vim lead implantation, there was unexpected ipsilateral worsening of right-hand tremor, and he was unable to engage a pen to paper or draw a spiral (similar to the pre-operative state). **D:** The left-hand tremor improved following right Vim DBS optimization. **E:** Following optimization of bilateral DBS, with an increase in left Vim amplitude, there was further improvement of tremor documented in both hands. *Created with BioRender.com*.

His presurgical examination revealed a moderate amplitude rest tremor, moderate-to-severe amplitude postural tremor, and severe amplitude intention and kinetic tremor in the upper extremities, with the right side being slightly more affected. Additionally, he exhibited a mild-to-moderate amplitude head tremor. The severity of his tremor was evident in his inability to write or to draw an Archimedes spiral with either hand prior to surgery (Figure 1A).

He underwent initial implantation of a left dual lead ventralis intermedius (Vim) and ventralis oralis (Vo) DBS system (Medtronic SenSight 33015). The decision to include Vo DBS was made as a potential adjunct to Vim DBS, given the severity of his tremor and his inability to engage a pen to a piece of paper during his examination. The left Vim DBS was programmed using a monopolar configuration with 10– C+, an amplitude of 1.3 mA, a pulse width of 90 µs, and a frequency of 135 Hz. This stimulation resulted in a significant improvement in the right upper extremity, including resolution of rest and postural tremor, reduction of intention tremor, kinetic tremor, and Archimedes (spiral-based) tremor to mild-to-moderate amplitude (Figure 1B). The patient was satisfied with improvement in activities of daily living and was content with the minimal current density settings after just a few programming sessions. Left Vo DBS was not needed, and he elected to reserve the use of this lead for potential future worsening of symptoms.

Seven months after the initial left-sided surgery, the patient underwent right Vim DBS implantation. Unexpectedly, this procedure led to an acute worsening of the previously optimized right upper extremity tremor (Figure 1C), even before stimulation was turned on. This resulted in a recurrence of the inability to engage a pen to paper, similar to his pre-operative state, suggesting a potential lesion effect. The patient did not experience any worsening of speech, swallowing, or walking following the right Vim DBS implantation.

## Clinical Dilemma

This case highlights the potential impact of implanting a second DBS lead when performing staged bilateral Vim DBS for severe essential tremor. The implantation of a second lead can result in unexpected changes in ipsilateral symptom response, which may manifest as either improvement or worsening of tremor control. While ipsilateral benefits are a desirable outcome, in rare instances, patients may experience an acute worsening of ipsilateral tremor following the second surgery.

This phenomenon presents a unique challenge for clinicians, as they must swiftly recognize, appreciate, and address the sudden loss of previously achieved tremor control in an ipsilateral limb after the contralateral DBS implantation. Effective management of this scenario requires a tailored approach to troubleshooting and potential reprogramming the DBS system to optimize the outcome.

## Clinical Solution

In managing this case, the initial step was to deactivate the left DBS lead and to focus on optimizing the newly implanted right Vim DBS lead (Figure 1D). This approach facilitated the assessment of the right Vim DBS effect on tremor without the confounding influence of the left DBS lead. The right Vim DBS was programmed using a monopolar configuration, with the optimal settings being 10– C+, an amplitude of 2.3 mA, a pulse width of 90 µs, and a frequency of 135 Hz. During the same initial programming visit, the left Vim DBS was reactivated without changing the stimulation parameters. As expected, the reactivation led to an improvement in the right upper extremity tremor, but also in the left upper extremity. The right-sided tremor was further optimized by carefully increasing the amplitude of left Vim stimulation from 1.3 mA to 2.2 mA, while maintaining the same pulse width and frequency.

It is noteworthy that although the right upper extremity tremor initially worsened after the right Vim DBS implantation prior to activation, the patient ultimately experienced better tremor control in both upper extremities after optimizing the new DBS lead through programming and through reactivating the old DBS lead (Figure 1E). This improvement was superior to the tremor control achieved with either left Vim (Figure 1B) or right Vim stimulation alone (Figure 1D). Moreover, we were able to further improve tremor control by fine-tuning the stimulation without inducing adverse effects such as motor or speech disturbances, which are common concerns when increasing DBS intensity.

## Gap in knowledge

Staged surgeries, where the leads are implanted on separate days, can offer several advantages. Some patients may achieve satisfactory results with a single lead, eliminating the need for a second surgery and its associated risks [1]. Additionally, if the first DBS lead results in side effects, staging the procedures can prevent the patient from undergoing a potentially unnecessary second surgery. However, when a second lead is required, its impact on side effects and symptoms can be highly variable, likely depending on both surgical factors, such as lead localization, and individual factors, such as unique fiber tract anatomy. While an ipsilateral improvement following the second DBS lead implantation is a desirable outcome and may aid in reducing the required stimulation, the potential emergence of side effects or acute ipsilateral worsening should not be overlooked.

In rare cases, implantation of a second lead contralateral to the first lead in staged bilateral thalamic DBS surgeries may present with a sudden loss of ipsilateral tremor benefit. Interestingly, the worsened tremor can often be recaptured by adjusting the programming settings of the initial lead. Counterintuitively, applying more stimulation to the initial lead may be the most effective approach to manage this situation, rather than reducing the stimulation intensity. The underlying mechanisms of this phenomenon remain poorly understood, and further research will be required to elucidate the complex interplay between bilateral DBS leads, lead localization, individual anatomy, and the resulting impact on tremor control as well as side effects.

## Expert Commentary

In staged bilateral Vim DBS, clinicians must ‘keep an eye on the first side’ and be vigilant in monitoring for both new onset side effects and for changes in tremor control on the previously treated side.

Stimulation-induced side effects, such as dysarthria and gait disturbance, are more common in bilateral Vim DBS as compared to unilateral cases [1,2,3,4,5,6,7]. Troubleshooting these side effects commonly begins with deactivating the device which will aid in the differentiation between microlesion and stimulation-induced effects. The DBS leads should then be tested individually to identify if a specific contact or side of stimulation may underpin the effect. If one lead is primarily driving the side effects, programming strategies can be focused on that lead. However, if both leads are contributing, programming evolves to become more complex, as reactivating the second lead may either improve or alternatively worsen symptoms. When stimulation-induced side effects occur, post-operative imaging is crucial and can be used to guide programming decisions, such as reducing current density or pulse width (Table 1).

**Table 1:** Vim DBS Side Effect Troubleshooting Guided by Lead Localization.


	DYSARTHRIA		MUSCLE CONTRACTION	ATAXIA	PARESTHESIA
		
**Lead position**	Medial	Lateral	Lateral	Ventro-medial	Posterior

**Structure**	Medial Vim	Internal capsule		Cerebello-thalamic input	Vc

**Common programming strategies**	Lowering amplitude or pulse width Changing electrode configuration (different contacts or bipolar configuration) Using interleaving or cycling				

**Specific strategies**	Using a combination of low frequency and high pulse width Choosing more dorsal contacts	Using segments to reduce lateral stimulation		Choosing more dorsal contacts	Using segments to reduce posterior stimulation


Vim, ventralis intermedius nucleus; Vc, ventral caudal nucleus.

**Figure 2:**
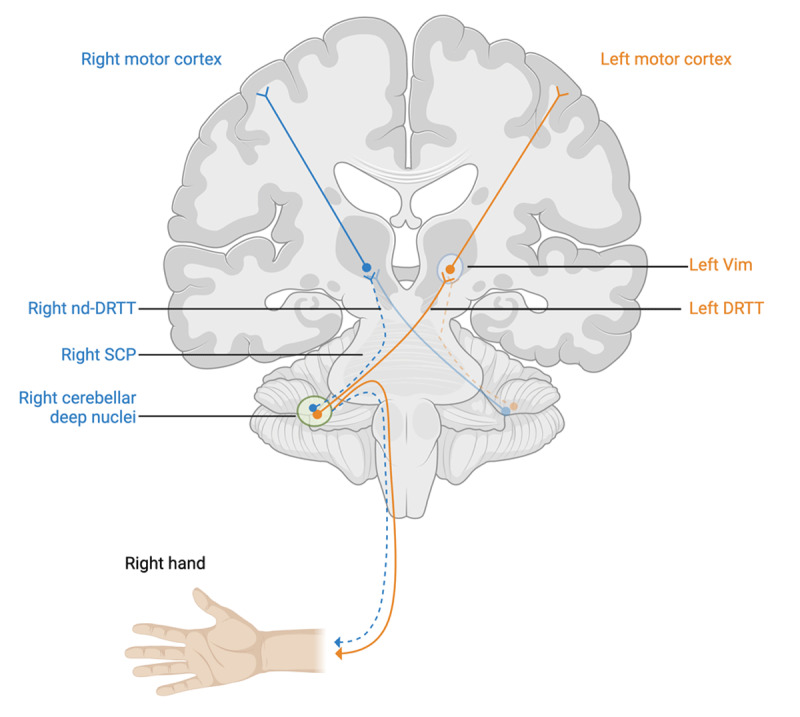
Dentatorubrothalamic tract (DRTT, solid lines) with non-decussating fibers (nd-DRTT, dashed lines). SCP; superior cerebellar peduncle. Vim; ventralis intermedius nucleus. *Created with BioRender.com*.

Clinicians should not only be attentive to side effects which may emerge following bilateral surgeries but also should appreciate and reassess for emergent worsening on the ipsilateral side. There is potential for ipsilateral tremor improvement, which may facilitate reduced stimulation and minimized risk of side effects. Our group and other academic expert centers have published on the phenomenon of ipsilateral tremor benefit resulting from unilateral Vim DBS [6,8,9,10]. Although many patients will not experience ipsilateral benefit, when present, it may be clinically significant, as evidenced by cases of inadvertent device deactivation [8,11]. Conversely, a sudden loss of benefit in a previously well-performing DBS lead should prompt immediate troubleshooting, as the suddenness of the presentation suggests a high likelihood that intervention can lead to improvement. When an ipsilateral benefit is lost, it should further pique the clinician’s interest in understanding the underlying neuroanatomical mechanisms. The ‘why’ ipsilateral question is likely rooted in the neuroanatomy (Figure 2).

A dysfunctional cerebello-thalamo-cortical circuit underpins the oscillatory hyperkinetic component of essential tremor [12]. Vim DBS has been shown to be a highly effective modulatory strategy when applied to this network [13]. Specifically, the dentatorubrothalamic tract (DRTT) connects the cerebellum to the contralateral Vim and is critical for Vim DBS benefits [13]. The DRTT originates in the deep cerebellar nuclei, decussates in the superior cerebellar peduncle, and connects to the red nucleus and thalamus (Figure 2) [14]. Interestingly, a small portion of the DRTT consists of non-decussating fibers (nd-DRTT), which can exert an ipsilateral motor influence [14,15]. Ipsilateral tremor changes could thus be hypothesized to occur through stimulation or lesioning of these nd-DRTT fibers, although other mechanisms may also be involved.

Cases of ipsilateral tremor worsening, particularly with pre-existing DBS leads, should raise suspicion for a potential microlesion or implantation effect. Systematically activating and deactivating each DBS lead, as performed in this case, is an appropriate management strategy. Differentiating between lesion and stimulation effects provides a solid foundation for further management. Deactivating the “old” DBS lead and focusing on optimizing the “new” DBS lead is a reasonable approach. Once optimized, the ipsilateral tremor outcome can be better characterized before reactivating and attempting programming of the second lead. While obtaining immediate post-operative imaging may not always be feasible, it can be critical for understanding the full clinical picture. Edema, hemorrhage, microhemorrhage along the DBS lead tract, and lead migration should all be considered as possibilities [16].

Multidisciplinary teams should always consider whether unilateral or bilateral DBS is appropriate, the timing of lead implantations (simultaneous or staged), and which side to prioritize; especially if unilateral or staged bilateral DBS is planned. Treatment decisions should account for the patient’s specific tremor characteristics and their expectations for improvement. Although bilateral thalamic DBS has been shown to provide greater reduction in appendicular and axial tremor compared to unilateral DBS, a few studies have found similar improvements in quality of life between the two approaches [2,3,17]. This of course raises the important pre-operative question as to whether two leads are always superior to one. For ET, many experts prefer single-sided, dominant-hand DBS. Moreover, staging lead implantations with an interval of weeks to months between sides is frequently recommended, particularly for elderly patients, to reduce the risk of adverse events.

In summary, this case highlights several key teaching points:

- Always reassess tremor on the previously operated side, as the outcome may be improved or worsened by the new lead.
- Sudden loss of benefit should trigger immediate troubleshooting, as there is a high likelihood of successful intervention.
- Sequential device deactivation, reprogramming, and clinical imaging are reasonable management strategies.
- Implantation and microlesion effects should be high on the differential diagnosis.
- Imaging can help narrow the differential diagnosis and may be particularly important in cases of hemorrhage or edema.
